# Toll-like Receptors 1, 3 and 7 Activate Distinct Genetic Features of NF-κB Signaling and γ-Protocadherin Expression in Human Cardiac Fibroblasts

**DOI:** 10.1007/s10753-025-02238-z

**Published:** 2025-01-20

**Authors:** Aditi Chaudhari, Camila Axelsson, Lillemor Mattsson Hultén, Victoria Rotter Sopasakis

**Affiliations:** 1 Department of Laboratory Medicine, Institute of Biomedicine, Sahlgrenska Academy, University of Gothenburg, Gothenburg, Sweden; 2 Department of Clinical Chemistry, Sahlgrenska University Hospital, Gothenburg, Sweden; 3 Wallenberg Laboratory, Department of Molecular and Clinical Medicine, Institute of Medicine, Sahlgrenska Academy, University of Gothenburg, Gothenburg, Sweden

**Keywords:** Toll-like receptors, Fibroblasts, Inflammation, Heart disease, NF-kB signaling, γ-protocadherins

## Abstract

Fibroblasts play a pivotal role in key processes within the heart, particularly in cardiac remodeling that follows both ischemic and non-ischemic injury. During remodeling, fibroblasts drive fibrosis and inflammation by reorganizing the extracellular matrix and modulating the immune response, including toll-like receptor (TLR) activation, to promote tissue stabilization. Building on findings from our prior research on heart tissue from patients with advanced coronary artery disease and aortic valve disease, this study sought to explore specific effects of TLR1, TLR3, and TLR7 activation on NF-κB signaling, proinflammatory cytokine production, and γ-protocadherin expression in cardiac fibroblasts. Human cardiac fibroblasts were exposed to agonists for TLR1, TLR3, or TLR7 for 24 h, followed by an analysis of NF-κB signaling, cytokine production, and γ-protocadherin expression. The activation of these TLRs triggered distinct responses in the NF-κB signaling pathway, with TLR3 showing a stronger activation profile compared to TLR1 and TLR7, particularly in downregulating γ-protocadherin expression. These findings highlight a potential role for TLR3 in amplifying inflammatory responses and reducing γ-protocadherin levels in cardiac fibroblasts, correlating with the enhanced inflammation and lower γ-protocadherin expression observed in diseased myocardium from patients with coronary artery disease and aortic valve disease. Consequently, TLR3 represents a potential therapeutic target for modulating immune responses in cardiovascular diseases.

**Supplementary Information:**

The online version contains supplementary material available at 10.1007/s10753-025-02238-z.

## Introduction

During ischemic conditions, oxygen depletion results in cardiac cell death. Conversely, in non-ischemic diseases, myocardial stress caused by factors such as hypertension, aortic stenosis, valve dysfunction, metabolic disease, or obesity leads to cardiomyocyte hypertrophy and chamber dilation. Both ischemic and non-ischemic heart diseases necessitate remodeling, including inflammation and fibrosis, during the acute phase to stabilize the injured area and maintain sufficient cardiac output [1]. However, inflammation must be tightly controlled, and the acute phase must be resolved for effective healing. Prolonged activity of innate immune cells can prevent resolution and contribute to cardiac dysfunction [2, 3].

Cardiac fibroblasts are essential for various processes in the heart, including maintaining extracellular matrix (ECM) homeostasis, providing a structural scaffold for cardiomyocytes, distributing mechanical forces through cardiac tissue, and mediating electric conduction [4]. Additionally, specific subsets of tissue fibroblasts contribute to the angiogenic response [5]. Following the acute inflammatory phase, the injured myocardium is replaced with a collagenous scar. This process involves activated cardiac fibroblasts, proliferating and transforming into myofibroblasts, and immune cells adopting a pro-fibrotic phenotype, both working in tandem [1]. A provisional matrix composed of fibrin and fibronectin is generated as the ECM is remodeled to facilitate cell proliferation and migration during fibrosis. Fibroblasts also play a crucial role as immunologic response cells to infection. They produce and regulate a variety of inflammatory mediators, including cytokines, chemokines, growth factors, antimicrobial peptides, and Toll-like receptors (TLRs) [6]. Consequently, fibroblasts have been proposed to act as crucial immunologic gatekeepers [6]. By expressing and responding to TLRs, these cells orchestrate a complex inflammatory response that is crucial for tissue repair and immune defense. However, dysregulation of this pathway can contribute to chronic inflammation and fibrosis, underscoring the importance of tightly regulated TLR signaling in cardiac health and disease.

Cardiac fibroblasts communicate with cardiac myocytes through various mechanisms. Communication through paracrine signaling involves secretion of various growth factors and cytokines, such as interleukin (IL)−1β, IL-6, tumor necrosis factor-α (TNF-α), and transforming growth factor-β (TGF-β) [7]. Indirect communication includes influencing the composition of ECMs in the heart by secreting collagen and fibronectin and activating matrix metalloproteinases, which, in turn, affect outside-in integrin signaling in cardiac myocytes [7]. Direct communication entails cell–cell interactions through connexins and cadherins [7]. These interactions influence heart function by altering cell-to-cell communication, cardiac myocyte proliferation, apoptosis, ECM composition, cell migration, and electrical conductivity, potentially leading to cardiac hypertrophy, remodeling, and heart failure [7].

Protocadherins (Pcdhs) are a subfamily of the cadherin superfamily, which are known to play crucial roles in cell adhesion and signaling. Although their primary research focus has historically been on their functions in the nervous system, emerging evidence suggests that Pcdhs also have significant roles in cardiovascular biology and heart disease. In this context, Pcdhs are implicated in various processes critical to heart development, maintenance, and pathology, for example the formation and maintenance of the intercalated discs, specialized structures that connect cardiomyocytes and ensure synchronized contraction of the heart muscle [8]. Whereas other cadherins have been implicated in inflammatory processes, Pcdhs are still vastly unexplored in this context.

The present study builds upon our previous findings in heart tissue from patients with advanced coronary artery disease and aortic valve disease, which demonstrated increased expression of TLR1, TLR3, and TLR7, along with elevated levels of their downstream signaling mediators. Additionally, these diseased myocardial tissues exhibited significantly decreased gene expression of γ-protocadherins (PCDHG) compared to control tissues [9, 10]. Consequently, this study aims to investigate the specific effects of TLR1, TLR3, and TLR7 activation on NF-κB signaling, proinflammatory cytokine expression and secretion, as well as PCDHG expression in cardiac fibroblasts.

## Methods

### Cell culture

Human cardiac fibroblasts from the ventricle of a 60 year old Caucasian male donor were purchased from PromoCell (cat# C-12375, lot# 450Z014.1, PromoCell, Heidelberg, Germany) and cultured at a seeding density of 12,000 cells/cm^2^ in 6 well TPP cell culture plates in Fibroblast Growth Medium 3 (cat# C-23025, PromoCell, Heidelberg, Germany). Cells were treated for 24 h with or without 100 ng/ml TLR1 agonist PamCSK4 (cat# NBP2-25,297, Novus Biologicals, Centennial, CO), 100 µg/mL TLR3 agonist Poly I:C (cat# NBP2-25,288, Novus Biologicals, Centennial, CO), 5 µg/mL TLR7 agonist R848 (cat# NBP2-26,231, Novus Biologicals, Centennial, CO) or 10 ng/ml lipopolysaccharide (LPS) derived from E.coli (Invivogen, San Diego, CA). The concentration of each agonist was chosen based on the maximum activation effect on TLR1, −3 and −7, respectively, as tested by the manufacturer and others [11, 12]. Dimetylsulfoxid (DMSO) (Sigma-Aldrich, Saint Louis, MO) was used as vehicle substance for control cells.

### RNA Extraction and Gene Expression Analysis

Cells were washed twice with ice cold PBS with Ca and Mg and lyzed in 350 µl RLT buffer (Qiagen, Valencia, CA) with DTT followed by RNA extraction of total RNA (including miRNA) using RNeasy Plus Mini kit (Qiagen, Valencia, CA).

For gene expression analyses of the NF-κB signaling pathway, the RT^2^ Profiler™ PCR Array Human NF-κB Signaling Pathway Plus (PAHS-025Y, Qiagen, Valencia, CA) was used. cDNA was synthesized with the RT^2^ First Strand kit (Qiagen, Valencia, CA) in a Gene Amp PCR system 9700 (Applied Biosystems, Foster City, CA).

For γ-protocadherin and microRNA gene expression analysis, real-time reverse transcription-PCR (RT-PCR) was performed. cDNA was prepared using a high capacity cDNA kit with random hexamer primers (Applied Biosystems, Foster City, CA). The TaqMan assays used are listed below. The qPCR reactions were performed with the QuantStudio 12 K Flex Real-Time PCR System (Applied Biosystems, Foster City, CA).

**Table:** 

Gene symbol	qPCR TaqMan assay	Manufacturer
MIR133A2	Hs04233528_s1	Thermo Fisher Scientific
MIR448	Hs04231583_s1	Thermo Fisher Scientific
MIR518C	Hs06627658_g1	Thermo Fisher Scientific
PCDHGA10	Hs00259371_s1	Thermo Fisher Scientific
PCDHGA11	Hs01107482_m1	Thermo Fisher Scientific
PCDHGA4	Hs00259326_s1	Thermo Fisher Scientific
PCDHGA5	Hs00259333_s1	Thermo Fisher Scientific
PCDHGA6	Hs00259344_s1	Thermo Fisher Scientific
PCDHGA7	Hs00259352_s1	Thermo Fisher Scientific
PCDHGA9	Hs00259365_s1	Thermo Fisher Scientific
PCDHGB1	Hs00251712_m1	Thermo Fisher Scientific
PCDHGC4	Hs00219044_m1	Thermo Fisher Scientific
PCDHGC5	Hs00260846_s1	Thermo Fisher Scientific

### Cytokine Measurements

After exposure to TLR agonists for 24 h, the media was collected and briefly centrifuged at 500xg for 5 min to remove cell debris. The media was aliquoted and frozen at −80 °C until further analysis. The cells were lyzed in 200 µl RIPA buffer (Sigma-Aldrich, Saint Louis, MO) supplemented with 1 × cOmplete EDTA-free Protease inhibitor cocktail (Roche, Basel, Switzerland), and the cell lysates centrifuged for 10 min at 12,000 rpm to remove cell debris. Total protein content was measured by the Bicinchoninic Acid method (ThermoFisher Scientific, Waltham, MA) and used for normalization calculations of the secreted cytokines.

Quantitative determination of interferon gamma (IFN-γ), interleukin (IL)−1β, IL-2, IL-4, IL-6, IL-8, IL-10, IL-12p70, IL-13 and tumor necrosis factor-alpha (TNF-α) in the cell culture medium was performed using an ultrasensitive multiplex electrochemiluminescence immunoassay (V-PLEX Proinflammatory Panel 1 Human Kit from Meso Scale Diagnostics, Rockville, MD). Intensity of the emitted light was measured according to the manufacturer's instructions on an MSD QuickPlex SQ120 plate reader (Meso Scale Diagnostics, Rockville, MD) and the concentration of secreted cytokines were normalized to the total cell protein content of each sample.

### Statistics

Statistical analysis of the RT^2^ PCR array data was performed with Qiagen’s Web-based PCR Array Data Analysis Software, available at www.SABiosciences.com/pcrarraydataanalysis.php. Briefly, data were normalized with an automatic selection of genes from the full plate. The CT values for these genes were geometrically averaged and used for the delta delta CT calculations. The level of significance was calculated using ttest and a *p*-value of < 0.05 was considered statistically significant. Multivariate analysis was carried out by principal component analysis (PCA) using Qlucore Omics Explorer 3.9 (Qlucore AB, Lund, Sweden), capturing the maximum variance in the data and projecting the data onto a lower-dimensional space to identify patterns, clusters and outliers.

Data for γ-protocadherin gene expression and for secreted cytokine levels were plotted as the mean and standard deviation (SD). The level of significance was calculated using one way ANOVA and GrapPad Prism software version 10 (Graphpad Software, San Diego, CA). A *p*-value of < 0.05 was considered statistically significant.

## Results

### TLR1, TLR3, and TLR7 Agonists Impact NF-κB-signaling Differently

**Fig. 1:**
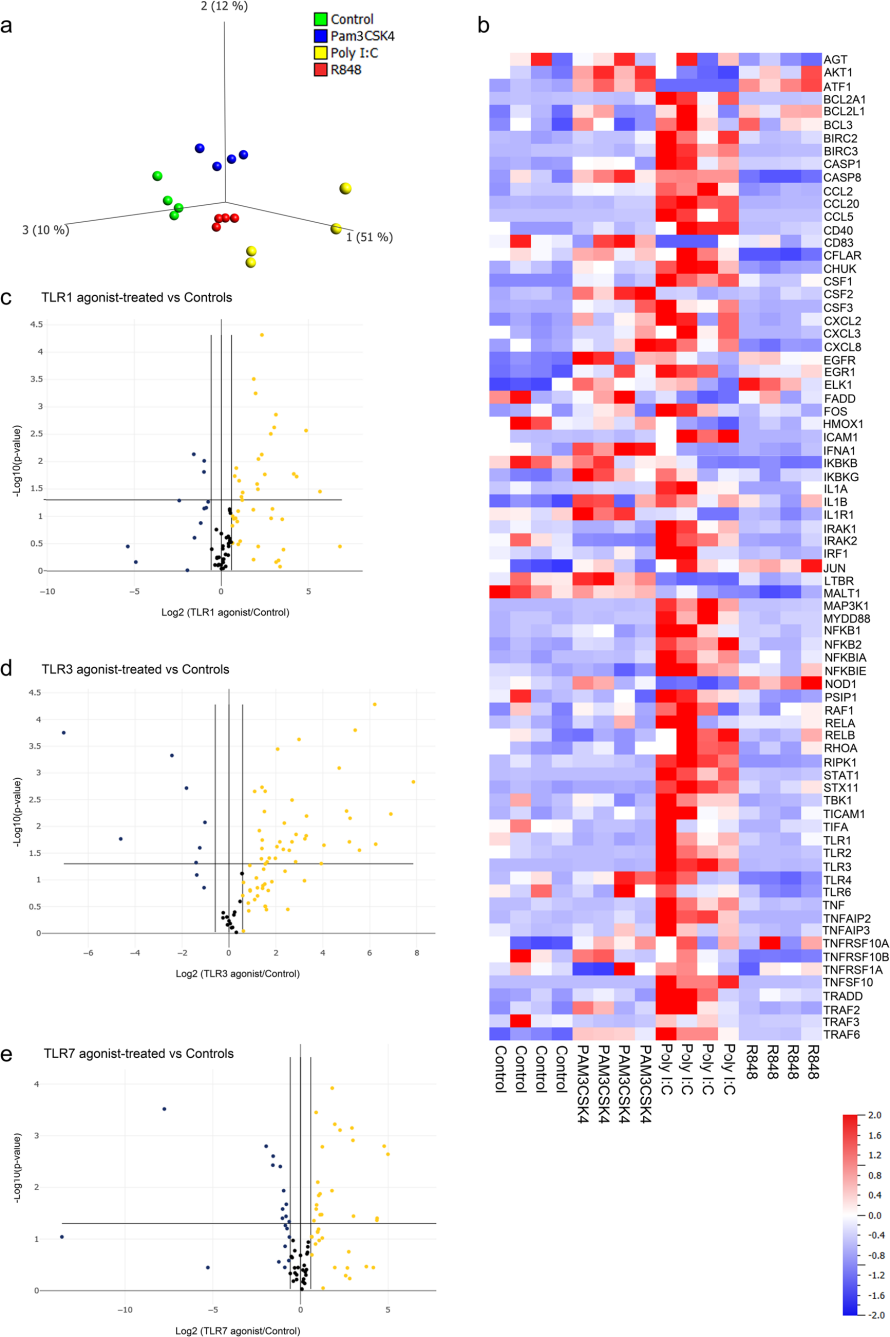
*Impact on NF-kκB signaling following activation of TLR1, TLR3 or TLR7 in human cardiac fibroblasts*. **a** PCA score plot (first three components) of 84 genes in the NF-κB pathway, showing a separation between cardiac fibroblasts treated with TLR1 agonist Pam3CSK4 (blue), TLR3 agonist Poly I:C (yellow) or TLR7 agonist R848 (red) and untreated control cells (green), (**b**) Heatmap of NF-κB pathway genes expressed in cardiac fibroblasts following treatment with Pam3CSK4, Poly I:C or R848 compared to untreated cells (controls), (C-E) Volcano plot displaying the difference in gene expression between cardiac fibroblasts treated with Pam3CSK4 (**c**), Poly I:C (**d**) or R848 (**e**) compared to untreated cells. *n* = 4, log2 fold change, *p*-value < 0.05 (ttest)

Human cardiac fibroblasts were treated with or without TLR1 agonist Pam3CSK4, TLR3 agonist Poly I:C or TLR7 agonist R848 for 24 h. The concentration of each agonist was chosen based on the maximum activation effect on TLR1, −3 and −7, respectively. 84 genes involved in the NF-κB signaling pathway were analyzed. Six genes were undetectable in all groups, CARD11, FASLG, IFNG, IL10, LTA, and TLR9 (Supplementary Table 1). Treatment with either of the three TLR agonists affected overall NF-κB signaling, but principal component analysis revealed distinct clustering of the treatment groups, indicating a difference in how the TLR agonists impact this pathway (Fig. 1a). This was confirmed when analyzing the gene expression data in more detail (Fig. 1b, Table 1 and Supplementary Table 1). Seven genes were upregulated by all three agonists; CSF1 (Pam3CSK4 = sevenfold change, Poly I:C = 26-fold change and R848 = eightfold change), EGFR (Pam3CSK4 = fourfold change, Poly I:C = threefold change and R848 = fourfold change), EGR1 (Pam3CSK4 = sixfold change, Poly I:C = sevenfold change and R848 = fivefold change), ELK1 (Pam3CSK4 = 20-fold change, Poly I:C = 17-fold change and R848 = 32-fold change), IL1R1 (Pam3CSK4 = twofold change, Poly I:C = threefold change and R848 = twofold change), MYD88 (Pam3CSK4 = fivefold change, Poly I:C = 119-fold change and R848 = eightfold change), and TRAF6 (Pam3CSK4 = fourfold change, Poly I:C = fivefold change and R848 = twofold change).

**Table 1:** Regulation of NFκB signaling by Pam3CSK4, Poly I:C or R848 in human cardiac fibroblasts compared to control cells, *n* = 3–4. Genes that were significantly changed for one or more agonist-treated sample are shown. n.s = not signficantly different compared to controls

Gene symbol	Fold change Pam3CSK4	*p*-value	Fold change Poly I:C	*p*-value	Fold change R848	*p*-value
AKT1	**29**	**0.003**	n.s		**21**	**0.04**
ATF1	**3.9**	**0.0006**	**0.01**	**0.0002**	**3.9**	**0.0006**
BCL2A1	n.s		**35**	**0.02**	n.s	
BCL2L1	n.s		n.s		**2.0**	**0.008**
BIRC2	n.s		**9.4**	**0.02**	**2.2**	**0.03**
BIRC3	n.s		**32**	**0.005**	n.s	
CASP1	n.s		**5.0**	**0.03**	**1.9**	**0.0004**
CASP8	**2.0**	**0.02**	**2.1**	**0.002**	**0.5**	**0.04**
CCL2	**1.8**	**0.01**	**4.1**	**0.02**	n.s	
CCL5	**2.2**	**0.04**	**76**	**0.02**	**0.00**	**0.0003**
CCL20	**4.2**	**0.03**	**74**	**0.00005**	n.s	
CD83	n.s		**0.04**	**0.02**	n.s	
CFS1	**7.2**	**0.003**	**26**	**0.0008**	**7.9**	**0.001**
CFS2	**5.0**	**0.007**	n.s		n.s	
CFS3	n.s		**15**	**0.05**	n.s	
CHUK	n.s		**2.9**	**0.002**	n.s	
CXCL2	**4.4**	**0.009**	**6.0**	**0.03**	n.s	
CXCL3	**51**	**0.04**	n.s		**21**	**0.04**
CXCL8	n.s		**2.5**	**0.01**	n.s	
EGFR	**4.3**	**0.03**	**2.6**	**0.03**	**3.5**	**0.0001**
EGR1	**5.6**	**0.02**	**7.2**	**0.01**	**4.8**	**0.0008**
ELK1	**20**	**0.02**	**17**	**0.02**	**32**	**0.002**
ICAM1	n.s		**7.2**	**0.05**	n.s	
IFNA1	**8.2**	**0.002**	n.s		n.s	
IKBKG	n.s		**0.42**	**0.03**	**0.26**	**0.002**
IL1A	n.s		**3.3**	**0.04**	n.s	
IL1R1	**2.3**	**0.04**	**2.7**	**0.002**	**1.9**	**0.02**
IRAK1	n.s		**0.4**	**0.047**	**0.5**	**0.004**
IRAK2	**0.5**	**0.01**	n.s		n.s	
IRF1	**0.3**	**0.007**	n.s		**0.5**	**0.03**
JUN	n.s		n.s		**0.6**	**0.02**
LTBR	**18**	**0.02**	n.s		**27**	**0.002**
MALT1	n.s		**0.3**	**0.002**	**0.6**	**0.046**
MAP3K1	n.s		**0.5**	**0.008**	**0.3**	**0.002**
MYD88	**5.0**	**0.00005**	**119**	**0.006**	**7.6**	**0.0007**
NFKB1	n.s		**6.4**	**0.02**	n.s	
NFKB1A	**1.7**	**0.02**	**6.4**	**0.003**	n.s	
NFKB2	n.s		**3.1**	**0.046**	n.s	
NFKBIE	n.s		**9.8**	**0.01**	n.s	
NOD1	n.s		**2.9**	**0.005**	n.s	
PSIP1	n.s		**0.2**	**0.0005**	**2.2**	**0.01**
REL	n.s		**2.7**	**0.02**	n.s	
RELB	n.s		n.s		**2.1**	**0.01**
STAT1	n.s		**4.2**	**0.0004**	**0.3**	**0.004**
STX11	n.s		**36**	**0.007**	n.s	
TBK1	n.s		**7.9**	**0.0002**	**2.3**	**0.03**
TIFA	n.s		**3.9**	**0.04**	n.s	
TIMP1	**0.5**	**0.02**	n.s		**0.6**	**0.04**
TLR3	n.s		**42**	**0.0002**	n.s	
TLR4	n.s		n.s		**0.5**	**0.01**
TNF	n.s		**47**	**0.03**	n.s	
TNFAIP2	n.s		**9.9**	**0.006**	n.s	
TNFRSF10A	**8.8**	**0.001**	n.s		**8.1**	**0.04**
TNFSF10	n.s		**232**	**0.001**	**3.5**	**0.01**
TRADD	n.s		**4.5**	**0.02**	**1.9**	**0.03**
TRAF2	n.s		**2.6**	**0.04**	n.s	
TRAF6	**3.6**	**0.0003**	**5.1**	**0.009**	**2.4**	**0.001**

*n.s* not signficantly different compared to controls

### Effect of TLR1 Agonist Pam3CSK4 on NF-κB-signaling

TLR1 agonist Pam3CSK4 treatment resulted in 20 genes that were significantly upregulated compared to control cells and three genes that were significantly downregulated (Fig. 1b-c and Table 1). The most upregulated genes were CXCL3 (51-fold change), AKT1 (29-fold change), ELK1 (20-fold change), and LTBR (18-fold change) (Table 1). The downregulated genes were IRF1 (0.3-fold change), TIMP1 (0.5-fold change) and IRAK2 (0.5-fold change) (Table 1). Of the 23 genes that showed a changed expression, three were uniquely changed by Pam3CSK4 and not by Poly I:C or R848: IFNA1, IRAK2 and CFS2 (Table 1).

### Effect of TLR3 Agonist Poly I:C on NF-κB-signaling

TLR3 agonist Poly:IC treatment resulted in 37 genes that were significantly upregulated compared to control cells and seven genes that were significantly downregulated (Fig. 1b and d and Table 1). Genes that exhibited particularly significant alterations included TNFSF10 (230-fold change), MYD88 (119-fold change), CCL5 (76-fold change), CCL20 (74-fold change) and ATF1 (0.01-fold change) (Table 1). Of the 44 genes that showed a changed expression, 19 were uniquely altered by Poly I:C and not significantly changed by Pam3CSK4 or R848 (Table 1), including BCL2A1 (35-fold change), BIRC3 (32-fold change), CD83 (0.04-fold change), CSF3 (15-fold change), syntaxin 11 (STX11) (36-fold change), TLR3 (42-fold change), and TNF (47-fold change).

### Effect of TLR7 Agonist R848 on NF-κB-signaling

TLR7 agonist R848 treatment resulted in 20 genes that were significantly upregulated compared to control cells and 11 genes that were significantly downregulated (Fig. 1b and e and Table 1). Upregulated genes included ELK1 (32-fold change), LTBR (27-fold change), AKT 1 (21-fold change), and CXCL3 (20-fold change). Downregulated genes included CCL5 (undetected following R848 treatment), IKBKG (0.26-fold change) and STAT1 (0.3-fold change). Of the 31 genes that showed a changed expression, four were uniquely changed by R848 and not changed by Pam3CSK4 or Poly I:C (Table 1): BCL2L1, RELB, JUN and TLR4.

### Secretion of Proinflammatory Markers by Cardiac Fibroblasts is Strongly Increased in Response to TLR1 and TLR3 Activation

**Fig. 2:**
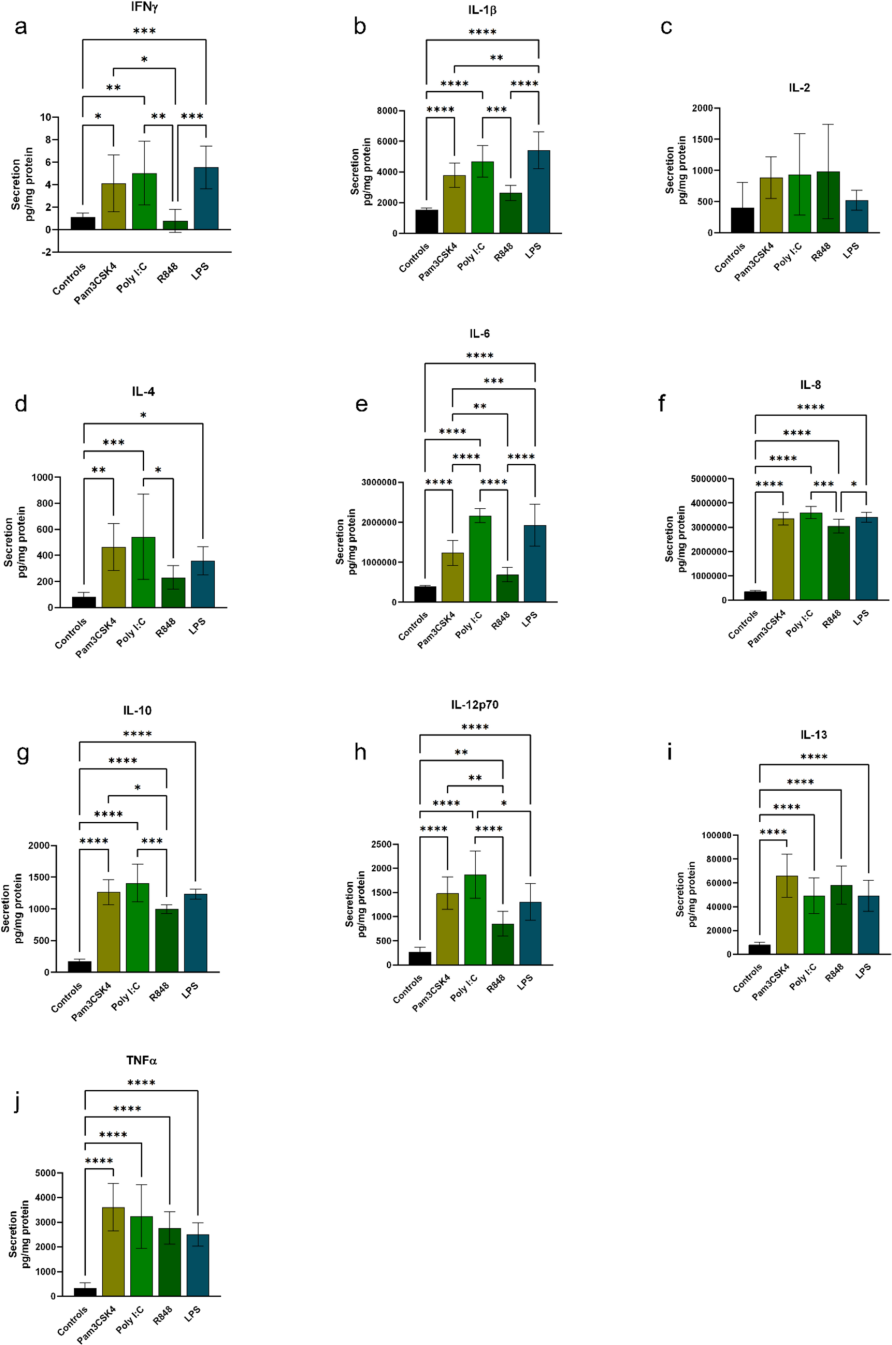
*Concentration of proinflammatory cytokines secreted from human cardiac fibroblasts following activation of TLR1, TLR3 or TLR7*. Cytokine concentrations were measured in conditioned medium following treatment of cardiac fibroblasts with TLR1 agonist Pam3CSK4, TLR3 agonist Poly I:C or TLR7 agonist R848 and compared to medium from untreated cells (controls). The TLR4 agonist LPS was used as a reference stimulant. The concentrations for each sample were normalized to the total protein content of that sample and expressed as pg/mg cell protein. Bars show data as the mean and standard deviation (SD). *n* = 7-8. * *p* < 0.05, ** *p* < 0.01, *** *p* < 0.001, **** *p* < 0.0001 (one way ANOVA). (**a**) IFNg = Interferon gamma, (**b**) IL-1b = Interleukin-1 beta, (**c**) IL-2 = Interleukin-2, (**d**) IL-4 = Interleukin-4, (**e**) IL-6 = Interleukin-6, (**f**) IL-8 = Interleukin-8, (**g**) IL-10 = Interleukin-10, (**h**) IL-12p70 = Interleukin-12 p70 (**i**) IL-13 = Interleukin-13, (**j**) TNFa = Tumor necrosis factor alpha

To evaluate if the increase in gene expression by TLR agonist simulation is mirrored by increased secretion of proinflammatory markers from the fibroblasts, we measured the concentration of ten proinflammatory cytokines in the cell culture media from fibroblasts treated with Pam3CSK4, Poly I:C or R848. Lipopolysaccharide (LPS) was included as a reference stimulant. IFNγ was secreted in very small amounts (~ 1 pg/mg protein for control cells), and increased 3–5-fold with Pam3CSK4, Poly I:C or LPS, whereas no changed was observed with R848 treatment (Fig. 2a). A similar pattern of secretion was observed for IL-1β, IL-4 and IL-6 (2–6-fold increase in secreted cytokines compared to controls) (Fig. 2b, d-e), although these markers were secreted at much higher levels compared to IFNγ, particularly IL-6 that reached levels of > 2μg/mg protein following Poly I:C treatment (Fig. 2e). Secretion of IL-8, IL-10, IL-12p70, IL-13 and TNFα was significantly increased by all TLR agonists (3–11 fold increase in secreted cytokines compared to controls) (Fig. 2f-j) as well as LPS and generally in very large amounts with IL-8 reaching levels of > 3μg/mg protein (Fig. 2f). IL-2 secretion was not significantly changed by any of the TLR agonists or LPS compared to control cells (Fig. 2c).

Taken together, secretion of proinflammatory markers by cardiac fibroblasts were generally strongly induced by TLR1 and TLR3 activation at levels similar to those observed by LPS stimulation, whereas TLR7 activation resulted in a more selective induction of cytokine secretion.

### TLR3 Stimulation with Poly I:C in Cardiac Fibroblasts Results in an Overall Decreased Gene Expression of γ-protocadherins

**Fig. 3:**
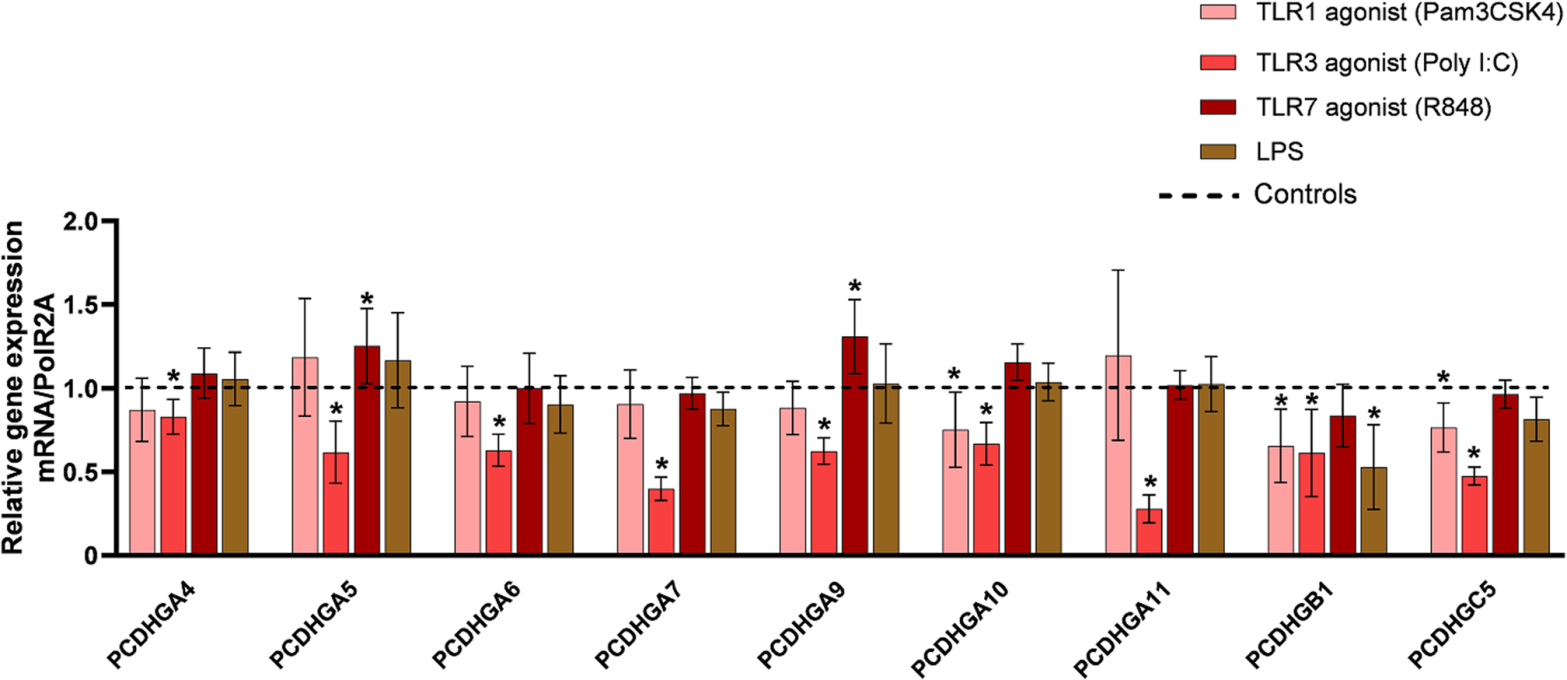
*γ-protocadherin gene levels in human cardiac fibroblasts following activation of TLR1, TLR3 or TLR7*. Bar graph displaying relative expression levels of γ-protocadherin genes. The dashed line indicates gene expression levels of untreated cardiac fibroblasts (controls), bars represent gene expression levels of fibroblasts treated with TLR1 agonist Pam3CSK4, TLR3 agonist Poly I:C or TLR7 agonist R848. Bars show data as the mean and standard deviation (SD). *n* = 8, *p-value < 0.05 (one way ANOVA)

We have previously reported that miR-133A/B- miR-448- and miR518C- signaling and associated *γ* -protocadherin (PCDHG) expression are markedly downregulated in cardiac tissue from patients with advanced coronary artery disease or valve disease [10]. These patients also showed upregulated expression of TLR1, −3, and −7 in the cardiac tissue compared to controls [9]. We therefore wanted to investigate if there is a direct effect of these toll-like receptors on miR-133A- miR-448-, miR518C- and PCDHG expression in cardiac fibroblasts. Lipopolysaccharide (LPS) was included as a reference stimulant. miR-133A, miR-448-, and miR518C expression was not detected in the cardiac fibroblasts (data not shown). Similarly, baseline PCDHGC4 expression was very low in these cells (data not shown). In contrast, expression of the remaining PCDHG genes investigated was significantly decreased by the TLR3 agonist Poly I:C by 17–72% (Fig. 3). TLR1 agonist Pam3CSK4 treatment significantly decreased the expression of PCDHGA10 by 25%, PCDHGB1 by 35% and PCDHGC5 by 24%, but did not change the expression of the other γ-protocadherin genes investigated (Fig. 3). The TLR7 agonist R848 surprisingly increased the expression of PCDHGA5 and PCDHGA9 by 25–30% without changing the expression of other γ-protocadherin genes (Fig. 3). The reference stimulant LPS significantly decreased the gene expression of PCDHGB1 without affecting the expression of any of the remaining γ-protocadherins.

Taken together, *γ*-protocadherins appear to be more potently regulated at gene level by TLR3 stimulation in cardiac fibroblasts compared to TLR1 or TLR7 triggers, independent of the micro-RNAs we previously found associated with decreased *γ*-protocadherin expression in the cardiac tissue of patients with heart disease.

## Discussion

The current study was based on our previous findings in heart tissue from patients with advanced coronary artery disease and aortic valve disease, showing that diseased myocardium displayed increased expression of TLR1, TLR3 and TLR7, increased expression of downstream signaling mediators of these receptors, as well as markedly decreased expression of γ-protocadherins compared to control tissue [9, 10]. Thus, we were interested in investigating possible direct and specific effects of TLR1-, TLR3- and TLR7-activation on NF-κB signaling, proinflammatory cytokine production as well as γ-protocadherin expression.

Toll-like receptors (TLRs) are broadly expressed across various mammalian cell types, including dendritic cells (DCs), macrophages, neutrophils, monocytes, lymphocytes, fibroblasts, epithelial cells, endothelial cells (ECs), and nerve cells [13]. Notably, each cell type expresses specific TLR subsets that play distinct roles in recognizing pathogen-associated molecular patterns (PAMPs) or damage-associated molecular patterns (DAMPs) and mediating the immune response [14].

Cardiac tissue consists of many different cell types and fibroblasts constitute a significant proportion of the total cell population in the heart. Estimates suggest that fibroblasts make up approximately 15–20% of the total cell number in the adult mammalian heart [4, 15–17]. This proportion, however, can vary depending on the species, age, and physiological or pathological conditions of the heart. Cardiac fibroblasts are vital to heart function, playing key roles in maintaining ECM homeostasis, providing a structural scaffold for cardiomyocytes, distributing mechanical forces throughout the cardiac tissue, and mediating electrical conduction [4, 18, 19]. While cardiac fibroblasts are known to express Toll-like receptors (TLRs) and respond robustly to proinflammatory stimuli [1], cardiomyocytes exhibit a comparatively muted response to such stimuli [20, 21]. In murine models, cardiomyocytes have been shown to be selectively responsive to activation by TLR2, TLR4, and TLR5, whereas activation by TLR3 and TLR7 did not result in the downstream expression of cytokines and chemokines (TLR1 was not investigated) [22]. Therefore, it is likely that fibroblasts significantly contribute to the increased expression of TLR1, TLR3, and TLR7 and their downstream signaling pathways we previously observed in the myocardium of patients with cardiac disease [9]. The data from the current study demonstrate that cardiac fibroblasts indeed respond to activation by TLR1, TLR3, and TLR7, leading to downstream NF-κB signaling. Interestingly, principal component analysis (PCA) revealed that these receptors activate distinct genetic features within this pathway. Specifically, we found that TLR3 regulate NF-κB mediators as well as γ-protocadherins at the gene level more potently and uniquely in cardiac fibroblasts compared to TLR1 and TLR7. Additionally, TLR1 and TLR3 activation strongly induced the secretion of proinflammatory markers by cardiac fibroblasts, reaching levels similar to those observed with LPS stimulation. In contrast, TLR7 activation resulted in a more selective induction of cytokine secretion. Notably, IL-10 was secreted in substantial amounts in response to all agonists, even though its gene expression was undetectable. This discrepancy might be explained by post-transcriptional regulation through microRNAs and RNA-binding proteins, which degrade or inhibit mRNA, reducing its levels without impacting existing IL-10 protein. Additionally, IL-10 may have strong protein stability and a long half-life, or might be efficiently translated, resulting in high protein levels even from minimal mRNA presence.

TLR1 forms heterodimers with TLR2 and is activated by triacylated bacterial lipoproteins, including the synthetic triacylated lipopeptide Pam3CSK4, which mimics the acylated amino terminus of proinflammatory bacterial lipopeptides [23]. Although TLR1 has not been directly linked to cardiovascular disease (CVD), one study demonstrated that TLR2 activates cardiac remodeling in a TLR1-dependent manner in a heart failure mouse model [24].

Activation of TLR7 has been shown to be essential for miR146a-5p-induced myocardial inflammation and cardiomyocyte dysfunction in mice [25], and both human and mouse cardiac tissue exhibit upregulated TLR7 gene expression following myocardial infarction (MI) [26]. Overactivation of TLR7 signaling induces severe hemorrhagic myocarditis in mice [27], while TLR7 deficient mice display reduced adverse cardiac remodeling and improved cardiac function [26]. Additionally, TLR7 knockout mice are protected against myocarditis following the induction of autoimmune myocarditis [28].

Specific ligands for TLR3 include viral double-stranded RNA (dsRNA) and its synthetic analogue polyriboinosinic (Poly I:C) [29]. Increasing evidence indicates that TLR3 plays a crucial role in the initiation and progression of cardiovascular diseases (CVDs). TLR3 has been implicated in mediating sterile pathologies in CVDs associated with immune responses, including pulmonary artery hypertension (PAH), atherosclerosis, myocardial infarction (MI), ischemia/reperfusion (I/R), and heart failure [30–32]. Mouse studies have demonstrated the role of TLR3 in cardiovascular disease and tissue repair. For example, TLR3 knockout mice or pharmacological inhibition of TLR3 in vivo prevented the development of aortic valve stenosis [33]. Conversely, TLR3 activation has been shown to be essential for repair and regeneration in damaged neonatal mouse hearts, promoting glycolysis and cardiomyocyte proliferation through downstream YAP1 signaling [34].

TLR3 is unique among the Toll-like receptors (TLRs), mediating its downstream signals independently of the adaptor protein MyD88, which is a central signaling mediator for all other TLRs. The potent molecular effects of TLR3 on NF-κB mediators and γ-protocadherins in cardiac fibroblasts may rely on this distinct MyD88-independent pathway. This pathway involves the recruitment of the adapter protein Toll-IL-1 receptor (TIR) domain-containing adapter (TRIF, also known as TICAM-1) and downstream association with receptor-interacting protein 1 (RIP1), TRAF3, and/or PI3K [35–38]. Indeed, angiotensin II-induced hypertension and myocardial hypertrophy in mice have been shown to depend on, and be regulated by, TLR3-TRIF signaling [39]. Furthermore, Tang et al. demonstrated that TLR3 mediates myocardial hypertrophy through increased binding to S-nitrosylated muscle LIM protein and RIP3 (receptor-interacting protein kinase 3) and the activation of the NLRP3 (NOD-like receptor pyrin domain containing 3) inflammasome [40]. However, TLR3 has been shown to signal through MyD88 in specific cases, such as during neuronal development, where TLR3 negatively regulates the expression of Disrupted in schizophrenia 1 (Disc1) in cultured neurons and mouse brain studies [41]. In our study, activation of TLR3 by Poly I:C led to a strong increase in MyD88 expression, suggesting that TLR3 may signal through MyD88 in cardiac fibroblasts, or alternatively, that MyD88 functions as a negative feedback mechanism, acting as an inhibitor of TLR3/TRIF activation, similar to findings observed in mouse corneal epithelium by Johnson et al. [42].

We have previously observed that patients with advanced coronary artery disease or aortic valve disease exhibited reduced expression of several γ-protocadherin (γ-Pcdhs) genes in myocardial tissue compared to control samples [10]. We hypothesized that this reduction was linked to the upregulated expression of TLR1, TLR3, and/or TLR7 in the same tissue samples. γ-Pcdhs are a subfamily of the cadherin superfamily, well-known for their roles in neural development and cell–cell adhesion. Whereas other cadherins have been implicated in inflammatory processes, Pcdhs are still vastly unexplored in this context. However, some studies suggest an involvement of Pcdhs in inflammatory responses, for example in PcdhgC3 KO brain microvascular endothelial cells, which showed an increased induction in IL-6 following oxygen/glucose deprivation or TNFα treatment compared to wildtype cells [43]. Furthermore, PCDHA4 levels were reduced in extracellular vesicles released from thrombin-activated platelets, while increased in platelet membranes [44]. Human monocyte-derived macrophages transfected with the S protein of SARS-CoV-2 resulted in increased IL-1β, IL-6, and TNFα protein levels, accompanied by a decreased gene expression of PCDHs [45]. Additionally, the possible role of Pcdhs in cardiac morphogenesis and cellular signaling during heart development further underscores a potential role in cardiomyopathy associated with inflammation [46].

Our data in the current study suggest that PCDHGs are more significantly regulated at the gene level by TLR3 stimulation in cardiac fibroblasts compared to TLR1 or TLR7 stimulation. This regulation occurs independently of miR-133A/B, miR-448, and miR518C that we previously found associated with decreased PCDHG expression in diseased myocardium. The absence of these miRNAs in fibroblasts suggests that their influence on PCDHGs pertains to other cardiac cell types, such as cardiomyocytes or endothelial cells. Thus, PCDHGs in fibroblasts appear to primarily be regulated by TLRs through mechanisms not involving miR-133A/B, miR-448, or miR518C. It is important to note that other mechanisms may also regulate PCDHG expression, e.g., hypoxia, oxidative stress, mechanical stress, extracellular matrix changes, and growth factors like TGF-β, which are particularly relevant in the context of cardiac remodeling and fibrosis. These aspects warrant further investigation in future studies.

In summary, our findings indicate that TLR1, TLR3, and TLR7 activate distinct genetic features of the NF-κB signaling pathway in cardiac fibroblasts, with TLR3 emerging as a more potent activator compared to TLR1 and TLR7, particularly in suppressing PCDHG gene expression. Our data support a role for TLR3 in cardiac fibroblasts in contributing to the enhanced inflammatory state and reduced PCDHG expression previously observed in the diseased myocardium of patients with advanced coronary artery disease and aortic valve disease. Thus, TLR3 represents a potential therapeutic target for modulating immune responses associated with CVD.

## Supplementary Information

Below is the link to the electronic supplementary material.

Supplementary file1 (XLSX 16 KB)

## Data Availability

No datasets were generated or analysed during the current study.
